# Round the Hospitals

**Published:** 1919-06-28

**Authors:** 


### ROUND THE HOSPITALS.

The annual meeting of the College of Nursing held on June 18 at Manchester was in all respects a very notable function. The choice of this locality for the gathering was justified by our excellent attendance. Many members came from London, some from Brighton, Truro, and every part of England and Wales, some even from the north of Scotland and from Ireland. Over 300 nursing sisters were present and the traditional hospitality of Manchester was well illustrated in the reception they received at the hands of the Local Centre in that town, and of the Lord Mayor and Lady Mayoress. The high educational ideas of the College were fitly recognised in the loan of the Chemistry Theatre at the University, conceded by Sir Henry Miers, who was present during the first part of the meeting. The greatest interest was displayed by members in the admirable organisation of the Manchester Centre and the announcement

**ROUND THE HOSPITALS**—*(continued).*

that Miss Sparshott had received the honour of the C.B.E. gave occasion for many hearty and sincere congratulations to her on the success of her labours.

---

Two novel points of considerable interest to nurses generally were brought before the meeting. Miss Cox Davies, in speaking of the development of the educational work, announced that the Council had it in mind to secure a Chair of Nursing at one of the women's colleges, with an expert professor, herself a trained nurse, in the Chair as lecturer on the selected subjects. There can be no doubt that such a step would tend more than anything else to advance the educational aims of the nursing profession.

---

The second matter was alluded to by Miss Cann, who asked Sir Arthur Stanley whether in the Registration Bill it would be possible to secure the term "nursing sister" for trained nurses in lieu of the word "nurse." The Chairman gave a sympathetic reply, stating it was a matter which might very well be taken up. He made the good suggestion that the proposal should be discussed first in the Local Centres, and in our judgment this should be done without delay all over the country. If petitions in favour of securing the term "nursing sister" for registered nurses were sent up to headquarters from all the local centres, there is every reason to believe this honourable title could be won for the exclusive use of trained nurses. We have the considered judgment of expert nurses on the other side of the Atlantic with us in declaring that the question of replacing the ambiguous word "nurse" by one which carries with it a recognised and indisputable status will be a vital factor in deciding whether registration will command the attention of all nurses qualified to come on the Register.

---

Great satisfaction has been caused to a wide circle of our readers, non-members of the College not excepted, by the announcement that two nurse members had been returned on the College Council. Their presence will lend weight to the Council, and we understand they are prepared to work hard. This is a clear indication that the system of voting adopted for these elections is beginning to work smoothly. We are disposed to think that a system of proportional representation which allows members a second choice after recording their votes for the candidates they most desire to see on the Council would be even more effectual by giving a value to every vote.

---

The Royal National Pension Fund for Nurses holds its annual meeting on June 26, the report, as usual, presents many points of interest. The number of nurses drawing annuities at the end of last year was 2,550, the average annuity amounting to a little over £26 10s. The premiums paid in amounted to over £94,000, and the total amount now invested amounts to £2,136,659. The withdrawals in these hard times continue to be numerous, and amounted to 891 policies, held by 803 nurses. We note that it has been decided to close the sick-pay branch so far as new entrants are concerned, though nurses already insured for sick pay will be allowed to continue their policies. The great strain on the Junius S. Morgan Benevolent Fund, which is in debt to the extent of £319, testifies to the severity of the crisis through which holders of small annuities are passing, and the unusual number of nurses requiring rest and convalescent aid. The Edith Cavell Homes have co-operated in several cases. If even the most thrifty members of the profession are in need, what of the rest?

---

Mr. Louis H. M. Dick, Secretary of the Royal National Pension Fund for Nurses, did admirable service for nurses by his telling evidence before the Royal Commission on the Income Tax. It is well known that no exemption, abatement, or relief from income tax is obtainable in the case of persons residing outside the United Kingdom, unless a certificate can be obtained that they live abroad for reasons of health. Among the nurses receiving annuities from the Royal National Pension Fund for Nurses there are 109 residing abroad, sixty-six in British Dominions, and forty-three in other countries. From their annuities, which range from a few shillings per annum to about £95, income tax is at present deducted at the rate of 6s. in the £. Thus the average pension of £27 becomes £18 4s. Mr. Dick made a powerful appeal for the redress of this harsh treatment towards women, who by the greatest thrift and self-denial during their working years had sought to assure their independence. He was able to show that the cost of granting relief from income tax to British subjects of small means residing abroad would be so small as to be almost negligible, and his case is so good and was so clearly presented that it is hardly possible he will not succeed in softening the heart of the Chancellor of the Exchequer.

---

Excessive indignation has been roused among nurses owing to the disclosures made by the Bishop of London at a meeting of the Diocesan Preventive Council at the Church House, Westminster. He asserted that in a verified statement now before the London County Council, an official had testified to the fact that seven women in the uniform of nurses had been seen lying about with soldiers in one of the open spaces in London, and the Bishop stated that it was unfortunately true that it was one of the commonest pretences for women to assume nurses' uniform. The nursing profession owes gratitude to the Bishop for thus boldly speaking out. A protected uniform is one of the imperative needs of the nursing service, and unless registration provides it, legislation will fall short of its due effect.

---

We record with pleasure a general rise in salaries at the Westminster Hospital. The assistant-matron's

**ROUND THE HOSPITALS—(*continued*).**

salary (formerly £40 to £60) is now £60 to £80. Sisters are to have £50 to £75 in place of £35 to £55. Staff-nurses in charge of departments are to have £45 to £60; in all cases the rate of increment is £5. Probationers receive £16, £24, £26, and the term of engagement is altered from four to three years. Increases in salary are announced also at Southwark Poor Law Infirmary, where the matron is to receive £130; first assistant-matron, £70 to £80; second assistant, £60 to £70; night superintendent, £55 to £60, plus £15 war bonus in all cases. It must be noted that we are still a long way from attaining the standard scale of minimum salaries recommended by the College Committee. Under that scale the matron of Southwark Infirmary would have £350 and her first assistant £130, while the matron of the Westminster Hospital would have £250 and her assistant £100.

---

SALARIES have undergone substantial increases at the Royal Infirmary, Sheffield. The home sister is to receive £85-£90; the ward sister and out-patients' sister, £65-£70; the night sister, theatre sister, and masseuse, £75; the assistant theatre nurse £45-£50. Probationers commence at £13 and rise to £18; third and fourth-year nurses receive £23-£33.

---

One by one the Poor Law Infirmaries throughout the country are coming into line in the matter of duty hours. The latest converts to the eight-hour day system are the King's Lynn Guardians who adopted the principle of a 56-hour week at a recent meeting. Unluckily a proposal for instituting a 48-hour week which would have given the nurses a day off once a week and a half day on Sunday was defeated. Still, considering that the nurses have been working 84 hours a week (which included meal-times, when, alas, they were liable to be called on duty), the 56-hour week is a real reform. The Guardians, who are disposed to make haste slowly after the manner of Guardians may rise to the one day in seven yet.

---

A PLEASING little ceremony took place at the V.A.D. Auxiliary Hospital, Haddington, N.B., on June 11. At the close of an enjoyable concert largely attended by those who have shown a practical interest in the hospital since its opening in 1914, Lady Baird, on behalf of her staff, presented Sister Kennedy with a handsome gold wristlet watch and a writing-case as parting gifts. Sister Kennedy, in reply, acknowledge her great indebtedness to the members of the V.A.D. who had so ably staffed the hospital. Colonel Thorpe of the Black Watch also spoke, and in moving a vote of thanks to the hospital staff paid a high tribute to the excellent work they had done. He said it was reputed to be one of the very best V.A.D. hospitals in Scotland.

---

The St. Marylebone Infirmary appears to be on the eve of radical alterations, the need for giving better training to the nursing staff being the immediate stimulus to long-needed reforms. The medical superintendent of the Infirmary has presented a report to the guardians reflecting on the efficiency of the medical work as carried on under the present system. With a wide range of cases they have the voluntary services of only one specialist, and they cannot claim to provide modern medical treatment. The supply of nurses is likely to fail unless drastic changes take place, since probationers and their relatives are better-informed than formerly about the conditions which constitute good training. A special staff of visiting consultants is deemed essential, and the first steps should be the engagement of a specialist in *x*-ray and electrical work. In the discussion which followed this able report it was elicited that nurses had been known to work thirteen and a-half and fourteen hours a day in the Infirmary. Nearly all the sisters had been called up, and they had the greatest difficulty in obtaining nurses, which is perhaps hardly to be wondered at. One of the lady guardians defended the training school, which she declared had a high standard. It is clear that no tinkering policy will serve the St. Marylebone Infirmary. A comprehensive reconstruction is imperatively to be demanded in the interests both of patients and nurses.

---

A CAPITAL sale was held in the grounds of the York County Hospital on June 14 in aid of that institution by Miss Steele and her nursing staff. The proceedings began with the presentation at the opening ceremony of medals to Senior Nurse A. Satterthwaite and Junior Nurse Elsie Tibbles. It was stated that the patients had co-operated with the nurses in producing the fine show of articles displayed for sale, and that one little blind girl had lent efficient fingers to the task. The proceeds amounted to about £67.

---

The managers of the Royal Infirmary, Edinburgh, gives, as usual, an interesting synopsis of the nurse training-school arrivals and departures in the annual report just issued. Out of 68 nurses who left between the months of October 1917 and 1918 no fewer than fifty-two joined the naval and military services—a splendid record which brought the total of those who have served to 402. The year appears to have been a very successful one, and we note with particular satisfaction that the daily percentage of nurses off duty, owing to illness and minor ailments, was reduced to 2.5, as against 3.1 the previous year; this figure includes those who suffered from influenza during the epidemic in July. The applications for training, though decreased in comparison with previous years, are still very good at 553. Many matrons in London would be glad if their average kept up at this level.

---

ABOUT half the county of Essex has been covered with nursing societies, and Dr. Thresh at the meeting of the Essex County Nursing Association

**ROUND THE HOSPITALS—*(continued)*.**

expressed his belief that within five years the whole county would be provided, and that there would in time be a nurse in every parish. They are starting a maternity home at Leytonstone, and the work of the Association shows a live and progressive tendency. The Bishop of Chelmsford, who made a good speech on the value to the hospitals of prevention, declared that all Church and philanthropic organisations ought to set an example by the way they paid people in their employ. He believed people should have a bit over a "living wage," so that they might not only live, but move and have their being. The Essex nurses begin at £80 and uniform as soon as their training is complete.

A NURSE who is in receipt of help from the Nation's Fund for Nurses writes to headquarters to express her thankfulness for the existence of the Fund. "Some nurses," she says, "object to being made out "paupers" by this Fund." She thinks they can hardly realise the position she and many others are placed in. She entered the profession thirty-seven years ago, has no home or parents living, and has never had a chance of saving. What is the prospect for a woman so placed when sickness or disablement overtakes her? Surely it is not the well-informed professional women who raise the cruel cry of pauper to a nurse thus rescued from distress through efforts made by nurses.

THE strike at the Clonmel Asylum of which tidings reach us has resulted in the escape of some fifty out of the 800 patients. In the absence of any detailed information about the circumstances which led up to the strike it is difficult to ascertain who are most to blame. On one hand, we hear that the Committee demurred to paying full wages to young and inexperienced attendants. On the other hand, the Committee appear to have ignored warnings previously conveyed to them, and to have attempted to bluff the situation without considered negotiations. They are reduced to imploring help from the military and police authorities. Such a complete administrative break-down calls for searching inquiry, and possibly for the transference of authority into more capable names.

THE surgeon who advertises this week for a "Nurse-Chauffeuse-Secretary" has obviously created a post which has come to stay. It implies first such a happy change of occupation as constitutes the ideal life. A busy hour or two in the surgery, morning and evening; books made up, correspondence attended to; then a delightful release to out-door avocations; long drives through country, or maybe town streets; long pauses before favoured patients' houses—all the life and movement of the world passing like a panorama before the amused vision; such is the idea. But we hope she has a boy to help to clean the car, and that night calls do not recur too frequently. There must be many nurses who possess the desired qualifications.

FOR the first time since August 1914 the London and South Western Railway has had a week in which no ambulance train was run. As a means of utilising the now idle ambulances the Bedfordshire War Pension Committee advise they should be fitted with massage appliances and sent round to minister to the disabled soldiers who in scattered areas are in some cases obliged to go twelve miles for half-an-hour's massage.

THE first woman to gain the American Distinguished Service Medal is Miss B. Macdonald, of the United States Reserve Nurse Corps. She was severely wounded during an attack on a British casualty station by German aircraft, and distinguished herself by coolness and resolution in sticking to her duties.

IN spite of the clouds a large and representative number of people gathered in the grounds of St. James's Palace at the garden party, in aid of the Nation's Fund for Nurses, on Tuesday, June 24. The scheme displayed an old English shopping fair, the booths arranged with originality and taste. The Viscountess Cowdray presided over a temptingly attractive stall of "Produce," filled with fruit, cakes and vegetables. "Soap and Scents," "American Candies" and "Smokes" each were tastefully displayed, and Miss Gladys Cooper and Miss Lilian Braithwaite were busily selling "Postcards and Posies," whilst near by the "Bijou Caledonian Market" drew great interest, in its arrangement of old crockery-ware. Strawberries and cream by "Mrs. Porges" were dispensed under the trees, and laughter and fun rippled around the ice-cream and hokey-pokey barrow. Here Mr. Gerald Du Maurier and Miss Mabel Russell, realistic in costume, did good business.

THE special features of the afternoon was a parade of the "Cries of London," girls and men in the picturesque dress of the Georgian period crying their wares complete with "Watercress," "Sweet Lavender," "Cherries," "Milk-O," and the rest. The *tout ensemble* made a charming picture. A series of English dances, by Miss Italia Conti's dancing children, was artistically performed, the dresses producing a rainbow effect of colour; nearly all the dancers were barefooted. Lady Lavery auctioned a kitten, it "going" at eight guineas. Good music was given by the band of H.M.'s Scots Guards, and an interesting exhibition of Highland dancing by the pipers of the Scots Guards. Tea, under the direction of Lady Fulton and numerous well-known lady helpers, was served in the marquee. The party was altogether a success. The whole-hearted efforts of the promoters secured interest, and the certainty of generous response by the visitors to their appeals in the needs of the nursing profession.

